# Bidirectional Reflectometry. Part I. A High Resolution Laser Bidirectional Reflectometer With Results on Several Optical Coatings

**DOI:** 10.6028/jres.080A.021

**Published:** 1976-04-01

**Authors:** Jack J. Hsia, Joseph C. Richmond

**Affiliations:** Institute for Basic Standards, National Bureau of Standards, Washington, D.C. 20234

**Keywords:** Barium sulphate, bidirectional reflectance, bidirectional reflectometer, black coating, gonioreflectometer, magnesium oxide, mu sulphur, reflectance, reflectometer, sodium chloride

## Abstract

A laser-source bidirectional reflectometer that is fully automated and has angular resolution on the order of one degree has been designed and built. The direction of incidence and viewing can be independently varied over an entire hemisphere except for directions more than 77.5° from the normal, and the two directions must be at least 2.5° apart. Bidirectional reflectances for 15 samples of black and white coatings are presented.

## 1. Introduction

The bidirectional reflectance distribution function (BRDF), *f_r_*, has been defined [1, 2]^1^ as a quantity that describes the detailed reflectance properties of a sample.

$$f_{r}\left(\theta_{i};\phi_{i};\theta_{r},\phi_{r}\right)=dL_{r}\left(\theta_{i},\phi_{i};\theta_{r},\phi_{r};E_{i}\right)/dE_{i}\left(\theta_{i},\phi_{i}\right)\left[sr^{-1}\right]$$

The angle *θ* is measured from the surface normal to the given direction, and *ϕ* is the azimuth angle from a reference direction on the sample to the given direction. The subscripts *i* and *r* refer to incident and reflected flux, respectively. *f_r_* is the quotient *dL_r_*(*θ_r_*, *ϕ_r_*)/*dE_i_*(*θ_i_*, *ϕ_i_*) of the radiance *dL_r_*, in the direction (*θ_r_*, *ϕ*_r_) due to irradiance *dE_i_* in the direction (*θ_i_*, *ϕ_i_*), over all possible combinations of the directions (*θ_i_*, *ϕ_i_*) of incidence and (*θ_r_*, *ϕ_r_*) reflection.

The reflectance of a material under any geometrical conditions of irradiation and viewing can be computed from *f_r_* by integrating over the solid angles of incidence and collection.

$$\rho \left(\omega_{i};\omega_{r};L_{i}\right)=\frac{\int_{\omega_{i}}\int_{\omega_{r}}f_{r}\left(\theta_{i},\phi_{i};\theta_{r},\phi_{r}\right)L_{i}\left(\theta_{i},\phi_{i}\right)\;\cos\theta_{i}\;\cos\theta_{r}\;d\omega_{r}\;d\omega_{i}}{\int_{\omega_{i}}L_{i}\left(\theta_{i},\phi_{i}\right)\;\cos\theta_{i}\;d\omega_{i}}$$

or, if *L_i_* is constant over the solid angle *ω_i_*

$$\rho \left(\omega_{i},\omega_{r}\right)=\frac{\int_{\omega_{i}}\int_{\omega_{r}}f_{r}\left(\theta_{i},\phi_{i},\theta_{r},\phi_{r}\right)\;\cos\theta_{i}\;\cos\theta_{r}\;d\omega_{r}\;d\omega_{i}}{\int_{\omega_{i}}\;\cos\theta_{i}\;d\omega_{i}}$$ (1.1)

Because of the importance of the BRDF in evaluating geometrically different types of reflectance, and in estimating errors due to flux losses in instruments designed to measure directional-hemispherical reflectance, equipment for its evaluation was designed and built. The average BRDF is computed from bidirectional reflectance by dividing by cos *θ_r_* Δ*ω_r_*, the projected solid angle over which reflected flux is collected in the measurement. Several criteria for such equipment were established before attempting to design the equipment. These included the following:

1. Incident radiation to consist of a highly collimated beam of monochromatic radiation, with provision for control of polarization.
2. Solid angle of collector to be small, on the order of one degree square, in order to achieve good angular resolution in the reflected beam.
3. Angles of incidence (*θ_i_*, *ϕ_i_*) and reflection (*θ_r_*, *ϕ_r_*) ^+^o be known to better than 1°.
4. Both angle of incidence and angle of reflection to be variable over nearly a complete hemisphere.
5. Increments between successive points of measurement to be variable.
6. Random error of measurement not to exceed 1 percent of the measured value, and bias not to exceed 1 percent of the measured value or 0.005 in reflectance, whichever is larger.
7. Automatic preprogrammed operation of the equipment, and on-line processing of the data with an electronic digital computer.

A 10-mW helium-neon laser with Brewster angle windows was selected as a source, to meet the requirements for a highly collimated, intense monochromatic incident beam. It can be operated at 0.6328, 1.15, and 3.39 *µ*m by use of cavity mirrors coated for operation at those wavelengths.

The requirement for automatic preprogrammed operation and computer processing of data is necessitated by the volume of data required. Evaluation of BRDF for points 1° apart over the hemisphere involves 400,000,000 data points. At 5° intervals, the number of data points is still over 1,600,000 points. Fortunately, most of the materials of interest are essentially isotropic in reflectance, that is, the bidirectional reflectance is essentially independent of the azimuth angle of incidence, *ϕ_i_*. For such materials, the directions of incidence for measurements at 5° intervals can be reduced from about 1250 to 18. Measurements in which the directions of incidence and reflection coincide, and those in which either direction is 80° or more from the normal, are difficult to make. By eliminating such measurements, the number of directions of incidence can be reduced to 16, and the total number of data points for complete evaluation to less than 20,000. By limiting the directions of reflectance to points lying in the plane of incidence, or in a few planes at fixed angles to the plane of incidence, the number of data points can be reduced to about 500 for each plane.

Due to a shift in priorities, part of the equipment has been disassembled and the instrument described here is no longer in operating condition. The preliminary results obtained in this study are being published to make available the data obtained, and to give others the benefit of our experience in designing and operating the equipment.

## 2. Bidirectional Reflectometer

**Figure 2.1:**
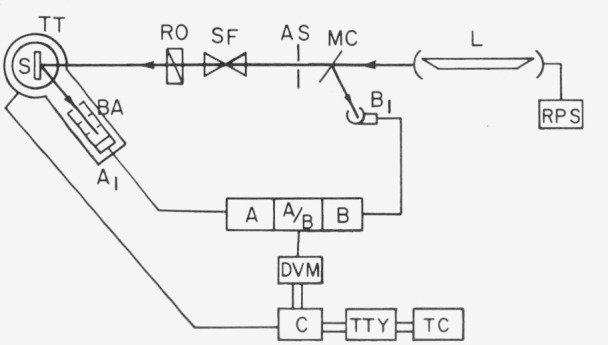
Schematic diagram of bidirectional reflectometer. RPS:Regulated power supplyMC:Mirror chopperSF:Spatial filterS:Sample holderBA:BafflesA:Amplifier AA/B:RatiometerC:ControllerTC:Time-shared computerL:LaserAS:Aperture stopRO:RotatorTT:TurntablesA_1_, B_1_:Detectors A and BB:Amplifier BDVM:Digital voltmeterTTY:Teletypewriter

As shown in figure 2.1, the beam was chopped by a mirror chopper, which directed the beam alternately along two paths. The beam reflected by the chopper went directly to a monitor detector. The beam passed by the chopper went through an aperture, spatial filter, polarization rotator and onto the sample. The sample was rotated about three axes by stepping motors to control the angles of incidence and viewing. The reflected flux was received by a baffled detector. The signals from the two detectors were separately amplified, and the ratio of the two signals was recorded by the data acquisition system.

### 2.1. Source

The source was a He-Ne continuous wave gas laser. Three pairs of interchangeable reflectors were provided to permit operation of the laser at 0.6328, 1.15, and 3.39 micrometers, respectively. The laser was used in the multimode confocal configuration. The multimode operation has the advantage of high amplitude stability. The confocal configuration makes alignment of the reflectors less critical. The laser beam was polarized and highly collimated with beam diameter about 2 mm. The current was supplied by a high voltage power supply. The nominal output of the He-Ne laser was given by the manufacturer as 10 milliwatts at 0.6328 *µ*m.

### 2.2. Mirror Chopper

The mirror chopper served two purposes: to direct the laser beam along two separate paths, and to modulate both of these beams so that phase-locked ac amplifiers could be used to amplify the signals, and thus reduce the random fluctuation of the two signals by reducing the effect of background. The double beam system [3], in which the signals from the two detectors were separately amplified, and the ratio of the two signals was used as a measure of the bidirectional reflectance, automatically compensated for the slight (about 2 percent) fluctuation in the laser output.

### 2.3. Aperture

An aperture with about 2 mm diameter was placed between the laser and the chopper. The aperture diameter was just larger than that of the laser beam to block light other than the main beam.

### 2.4. Spatial Filter

The spatial filter consisted of two lenses and one 20 *µ*m diameter pinhole. The pinhole could be adjusted horizontally and vertically by means of micrometer screws. The first lens focused the collimated laser beam onto the pinhole and the second lens converted the diverging beam from the pinhole into a collimated beam. The purpose was to further filter out the scattered light, and produce a highly collimated beam.

### 2.5. Rotator

The rotator unit contained two quartz, quarter-wave plates. The first plate could be rotated manually so that its plane of retardation could be set at 45 degrees from the plane of polarization of the laser, to produce a circularly polarized beam. The light beam, after passing through the first plate, was found to have 1.5 percent polarization. The second quarter-wave plate could be used to convert the circularly polarized beam back to a plane polarized beam. The plane of polarization could be varied by rotation of the second quarter-wave plane, which was accomplished by means of a stepping motor.

**Figure 2.2:**
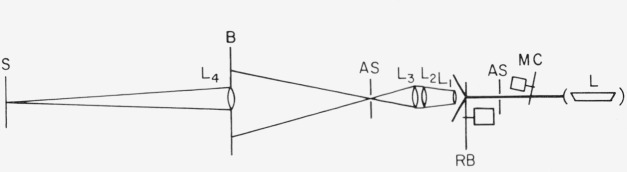
Schematic diagram of unpolarized laser light source arrangement. L:LaserAS:Aperture stopRB:Rotating blade with drafting film; rotation speed 1800 rpm with motor shaft to light beam distance of 7 cm.L_1_, L_2_, L_3_, and L_4_:LensesB:BaffleS:SampleMC:Mirror chopper

An attempt was made to generate an unpolarized light beam from the polarized laser beam. A blade made of K & E Herculene drafting film was inserted in the light beam after the aperture and rotated at 1800 rpm. This is an optically inhomogeneous film that transmits a large fraction of the incident beam. Because of the finite size of the inhomogenieties in the material, the plane polarized and well-collimated laser beam is broken up into a diffuse beam with more-or-less random polarization in which the direction of the plane of polarization varies with position in the field. By rotating the film, these variations are constantly changing at a rapid rate, and the time-averaged residual polarization over the field is uniform not only from point to point, but for the same point from time to time. Lenses were then used to focus the beam onto the sample as shown in figure 2.2. The light beam, after passing through the rotating blade, was found to have 20 percent residual polarization.

### 2.6. Sample Holder and Turntable

The sample holder was made of aluminum alloy with a 38.1 mm diameter open space in the center. The front side tapered 10° toward the edge of the opening and was painted with matte black paint. The back side of the holder had three protrusions against which the sample surface was clamped. The three protrusions were located on a circle of 63.5 mm diameter. The axis (*A*) of rotation of the holder was in the sample-surface plane formed by the tips of these three protrusions. This axis (*A*) was normal to and rotated about a horizontal gimbal axis (*B*). The sample holder and gimbal were also mounted on a turntable which rotated around a vertical axis (*C*), as shown in figure 2.3.

The relations of the azimuthal and polar angles relative to the sample surface with angles about these three axes will be discussed in section 3.

### 2.7. Stepping Motors and Control

The sample holder was rotated about each of the three respective axes by a separate stepping motor, through a motor controller. Instructions to the control could be typed manually on a Teletype, fed into the Teletype as pre-punched paper tape, or supplied through an acoustic coupler by an on-line time-shared computer. Rotation about the vertical axis (C) was controlled to 0.045° (200 steps/rev. gear ratio 40/1), and that about the other axes (*A* & *B*) to 0.1° (48 steps/rev. gear ratio 75/1).

### 2.8. Detectors

Two lead sulfide detectors were used, one to monitor the laser beam, and the other to measure the light reflected from or incident on the sample. Both were 10 × 10 mm in size, and were operated with a 180 V dc bias. The sample detector was mounted on an arm attached to a turntable so that it could be rotated about the vertical axis *C*, in the horizontal plane of the horizontal axis *B*, at a distance of 940 mm from the intersection of axes *B* and *C*, as shown in figure 2.3. The detector thus subtended a plane angle of 0.61°, or a solid angle of 1.132 × 10^−4^ sr, at this point of intersection. Baffles, with apertures selected to confine the field of view of the detector to an area about the point of intersection slightly larger than the exposed area of the sample, were placed in front of the detector to block scattered light from reaching the detector.

### 2.9. Averaging Spheres

Two averaging spheres, 76.2 mm in diameter, were used; one in front of the monitor detector, and one in front of the sample detector when measuring incident light. The inside of each sphere was coated with barium sulfate. Each sphere was so constructed that the area irradiated by the beam being measured was not viewed by the detector. The transmittance (or efficiency) of the sample detector averaging sphere was measured, using the double-sphere method, and found to be 0.72 percent. The calculated transmittance was 0.82 percent.

### 2.10. Double-Detector and Amplifier System

The double-detector system was used to make the measurement independent of the output level of the laser. The laser beam was chopped by the mirror chopper at 33.9 Hz. During the first half of each cycle, the beam was incident on the sample. During the second half, the beam was reflected by the chopper blade into a small averaging sphere. The radiance of the averaging sphere wall was measured by the monitor detector, whose signal was then fed into a phase sensitive synchronous preamplifier and amplifier. The signal from the sample detector was amplified by an identical amplifier. The outputs of the two amplifiers were then fed into a ratioing multiplexer whose output was proportional to the ratio of the two signals. The resulting signal was then fed into a digital voltmeter.

### 2.11. Data Acquisition and Data Processing

The signal from the digital voltmeter was printed by the Teletype. In our experiments the stepping motors were controlled by the controller which accepted commands from the Teletype through pre-punched paper tape. During the scan, each time that the stepping motors finished their rotation, the controller signaled the acquisition system to record ten data points. The signal from the digital voltmeter was printed by the Teletype and also fed into the on-line time-shared computer to process the data.

## 3. Azimuth and Polar Angles

The reflectometer controls the directions of incidence and viewing in terms of the angular position of the sample relative to the three axes of rotation of the sample holder. In order to convert the coordinates of these directions from the instrument angles into the more common polar and azimuth angles of the directions of incidence and viewing, relative to the sample normal and a fixed azimuth reference on the sample, it is necessary to transform the axes from those of the instrument to those of the sample.

The instrument axes are *A*, *B*, and *C*, and the corresponding angles about the respective axes are *a*, *b*, *c*. The pairs *A* and *B* and *B* and *C* are each mutually perpendicular, and all three meet at the origin, which lies in the plane of the sample surface. The *C* axis is vertical, and its positive direction is up from the origin; the *B* axis is horizontal, rotates about the *C* axis and always coincides with the direction of viewing. Its positive direction is from the origin to the detector. The *A* axis is normal to and rotates about the *B* axis. Its positive direction is from the origin to the stepping motor that rotates the sample about that axis.

Angle *ϕ*_0_ is the angle between the positive *A* axis and the fixed azimuth reference mark on the sample. This angle remains fixed once the sample is mounted in the sample holder. Angle *a* is measured about the *A* axis from the positive *B* axis to the sample normal. Angle *b* is measured about the *B* axis from the positive *C* axis to the positive *A* axis. Angle *c* is measured about the *C* axis from the direction of incidence to the positive *B* axis. Each angle is positive in the counterclockwise direction, when viewed from the positive direction of the respective axis of rotation toward the origin.

**Figure 3.1:**
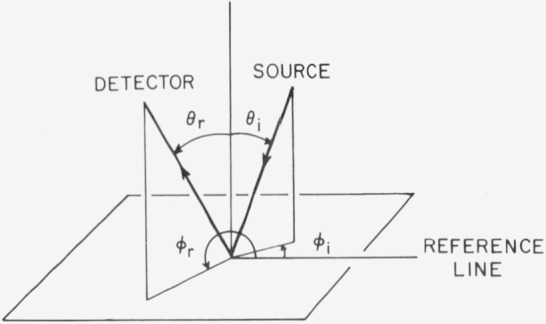
Coordinate system related to sample surface.

The desired parameters are *θ_i_*, *ϕ_i_*, *θ_r_*, *ϕ_r_*. (See fig. 3.1.)

*θ_i_* is the polar angle between the sample normal and the direction of incidence.
*ϕ_i_* is the azimuth angle between the reference line on the sample surface and the direction of incidence.
*θ_r_* is the polar angle between the sample normal and the direction of viewing.
*ϕ_r_* is the azimuth angle between the reference line and the direction of viewing.

The laser, and hence the direction of incidence, remains fixed. The detector rotates about the sample in the horizontal plane, and the direction of viewing always coincides with axis *B.*

The azimuth angle, *ϕ_r_*, between the reference line and the direction of viewing remains fixed once the sample is mounted in the sample holder. During design of the sample holder inclusion of provision for rotating the sample in its own plane was considered. Since most of the materials of interest are essentially isotropic,. equipment to provide such rotation is complex and somewhat bulky, and the space available for such equipment is small, it was decided that inclusion of this equipment was not justified.

The sample holder permits measurement of bidirectional reflectance at all angles of incidence where *θ_i_* ⩽ 80° and the direction of incidence is not closer than 2.5° to the direction of viewing, and at all angles of viewing where *θ_r_* ⩽ 80° at a single value of *ϕ_r_*. Since the beams of incident and reflected flux are of very small angular size, no error is introduced in the measured reflectance by interchanging the directions of incidence, *θ_i_*, *ϕ_i_* and reflectance, *θ_r_*, *ϕ_r_*. In this way it is possible to obtain data over nearly a complete hemisphere of angles of viewing for all angles of incidence, *θ_i_*, within a fixed azimuth plane. The location of the fixed azimuth plane can be changed, if desired, between series of measurements by manually rotating the sample in the holder.

When angles *a*, *b*, and *c* are zero, the *B* axis will coincide with the sample normal, the direction of incidence and the direction of viewing, and the *C* and *A* axes will coincide. As the sample is rotated about the several axes, its normal will move away from its zero position as follows: with *a* = *b* = 0, the normal will move from its zero position by angle *c*; as *b* is changed, the plane containing the sample normal rotates about axis *B* by angle *b*; when *b* = *c*= 0, the sample normal moves away from its zero position by angle *a.*

Since the direction of viewing coincides with the *B* axis, its direction is independent of angles *b* and *c*.

$$\theta_{r}=a.$$ (3.1)

For the same reason, *ϕ_r_*, the azimuth angle of the direction of viewing will be independent of angles *a*, *b*, and *c*.

$$\phi_{r}=90{}^{\circ}-\phi_{0}.$$ (3.2)

The equations for angles *θ_i_* and *ϕ_i_* are more complex. Several equations can be set up relating *θ_i_* to *a*, *b*, and *c*. When *b* = 0, *θ_i_* = *c* + *a*. When *b* = 0 and *a* = −*c*, *θ_i_* = 0. When *c = a* = 0, *θ_i_* = 0, regardless of *b*. When *b* = 90°, cos *θ_i_* = cos *a* cos *c*. When *a* = 0, *θ_i_*= |*c*|. Hence,

$$\cos\;\theta_{i}=\cos\;a\;\cos\;c-\sin\;a\;\cos\;b\;\sin\;c.$$ (3.3)

Equations can also be derived relating *ϕ_i_* to *a*, *b*, *c*, *ϕ*_0_ and *θ_i_.* When *θ_i_* = 0, *ϕ_i_* is indeterminate. When *a* = 0, *ϕ_i_* is independent of *c* and *θ_i_*, and *ϕ_i_* = [(*c*/|*c*|) · 90°] − *b* − *ϕ*_0_. When *b*= (*c/* |*c*|) · 90°, *ϕ_i_* = − *ϕ*_0_. The final equations are:

$$\begin{array}{c} \;\phi_{i}=\phi_{0}^{\prime }-\phi_{0}\\ \cos\;\phi_{0}^{\prime }=\frac{\sin\;b-\sin\;c}{\sin\;\theta_{i}}. \end{array}$$ (3.4)

**Figure 3.2:**
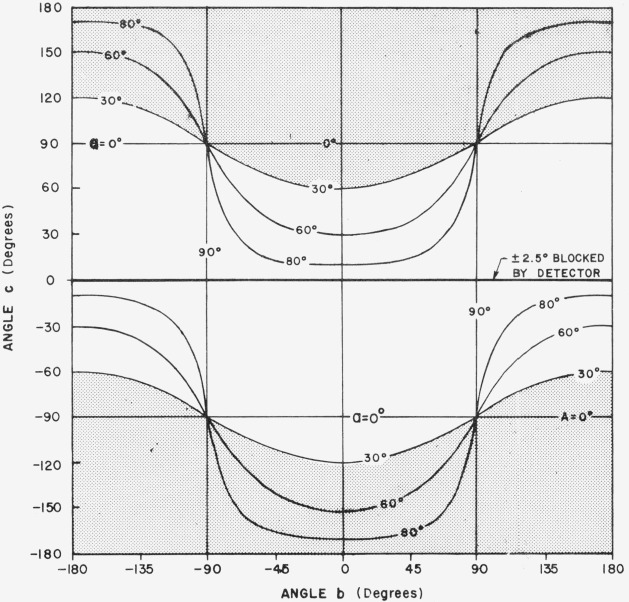
Physically impractical conditions of angles *a, b*, and *c*, indicated by shaded portion.

**Figure 3.3:**
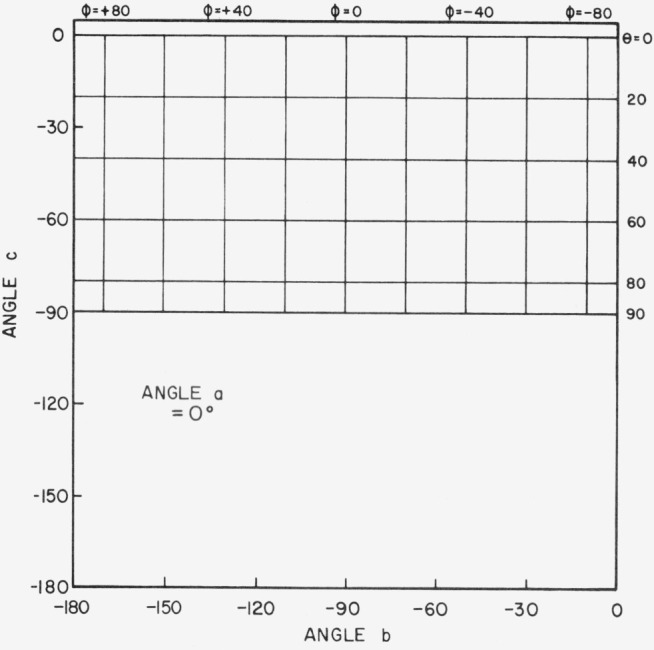
Relations between θ, ϕ and *b*, *c* when *a* = 0 degrees. *θ_r_ = a*, *ϕ_r_ = +* 90°, *θ_i_ = θ*, *ϕ_i_ = ϕ.*

**Figure 3.4:**
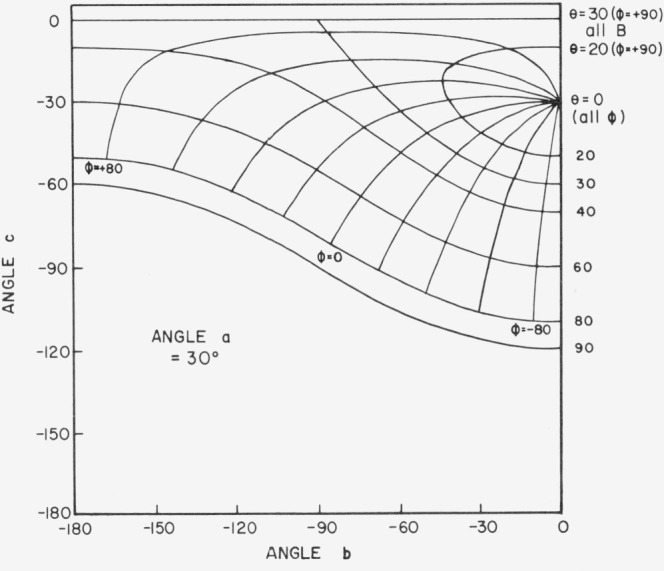
Relations between θ, ϕ and *b*, *c* when *a* = 30 degrees. *θ_r_=a*, *ϕ_r_*= + 90°, *θ_i_ = θ*, *ϕ_i_* = *ϕ*.

**Figure 3.5:**
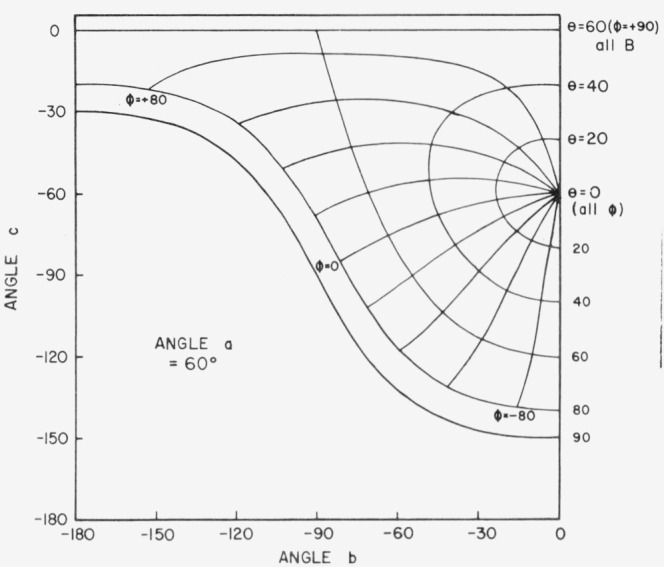
Relations between θ, ϕ and *b*, *c* when *a* − 60 degrees. *θ_r_ = a*, *ϕ_r_* = +90°, *θ_i_ = θ*, *ϕ_i_* = *ϕ.*

**Figure 3.6:**
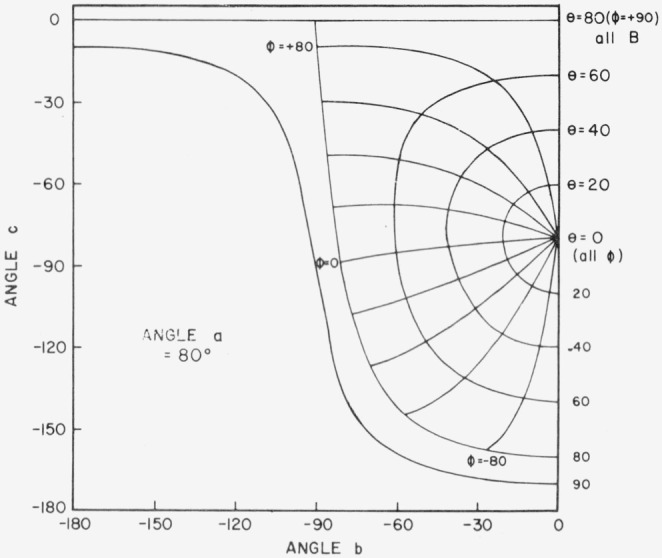
Relations between θ, ϕ and *b*, *c* when *a* = 80 degrees. *θ_r_ = a*, *ϕ_r_* = +90°, *θ_i_* = *θ*, *ϕ_i_ = ϕ.*

Figure 3.2 shows the physically impractical conditions of angles *a*, *b*, and *c*. When *a* = 30°, for example, and *b* and *c* lie in the shaded area of the figure, the incident beam will be on the back of the sample. Only one quarter of the possible combinations of *b* and *c* in figure 3.2 is enough to give all the polar and azimuth angles needed. Figures 3.3, 3.4, 3.5, and 3.6 show the relations between *θ* (i.e., *θ_i_*), *ϕ* (i.e., *ϕ_j_*), and *b*, *c* when *a* = 0°, 30°, 60°, and 80° respectively, *ϕ_r_* equals 90° when *ϕ*_0_ = 0. Thus when *ϕ_i_* = − 90°, the measurement is made in the plane of incidence.

## 4. Alignment

Alignment of the optical system involved the following steps: (1) leveling the laser beam to establish a level plane of reference for alignment of other optical elements; (2) leveling the sample holder and centering it in the laser beam; check to see that axis *C* is vertical, axis *B* is horizontal and in a horizontal plane through the laser beam, axis *A* is perpendicular to axis *B* and in a vertical plane through axis *C*, and that the three axes intersect at the point where the laser beam hits the sample; (3) establish the zero angular position about each axis; (4) align detector and detector baffle; and (5) position and align other optical elements: chopper, spatial filter, polarization rotator and aperture.

### 4.1. Leveling Laser Beam

Since the laser beam remained fixed in position, and served as the reference for alignment of the rest of the optical system, it was carefully leveled. A high-sensitivity machinists’ level was used to level the base on which the laser tube is mounted, which approximately leveled the beam. A surveyor’s level was then set up so that, when level, its crosshairs were in the horizontal plane containing the laser beam. The level was then sighted in turn on the two bright spots produced by the front and rear laser beams on the end walls of the laboratory, about 5 m apart. The level of the laser was adjusted until the crosshairs of the level telescope were well centered on each of the two bright spots. The level plane thus established became the reference plane for alignment of the other elements of the system, and the level was left in place until alignment was completed.

### 4.2. Positioning Sample Holder

The sample holder consisted of two separate parts, which were mounted on a different table from that on which the laser was mounted. Part one consisted of two rotary milling machine heads mounted one above the other so that their axes of rotation coincided. Two arms, each about 110 cm long, were attached to opposite sides of the bottom rotary head. One supported the detector and its baffles, and the other supported counterweights, so that the entire rotating head was approximately balanced. The upper rotary head supported part two, which was a mount for a horizontal shaft (axis *B*) with a yoke attached to one end holding a second shaft (axis *A*) normal to the first shaft.

Part one of the sample holder was clamped in place on the supporting table, which was of such height that the center of the sample, when the sample is mounted in the holder, would be approximately centered on the laser beam. Part two of the sample holder was placed in position, and a card with crossed vertical and horizontal lines was centered in the sample holder. The table was adjusted until the laser beam was centered on the crossed lines. A small graduated cylinder was placed near the end of the detector support arm, and filled with water until the meniscus was in the reference plane. The surveyor’s level was sighted on the meniscus, as the arm was rotated about the *C* axis, and the level of the table was adjusted until the meniscus remained in the reference plane at all positions of the arm. This showed that the *C* axis was vertical. The position of the laser beam was again checked on the crossed lines, the card was removed and the laser beam was found to be well centered on the end of the horizontal shaft (*B* axis). A plane mirror was mounted in the sample holder, angle *c* was set at about 40°, and angle *a* was adjusted until the bright spot on the wall formed by the laser beam after reflection from the mirror, remained stationary while angle *b* was varied from 0° to 180°. This showed the sample to be normal to axis *B.* Angle *c* was then changed until the laser beam was reflected back along its path, and the sample holder was shimmed until the sample was normal to the laser beam. The plane mirror was replaced with the card with crossed lines, and the card was adjusted until the laser beam was centered on the crossed lines. Angle *b* was varied from 0° to 360° to check that the cross was at axis *B.* The position of the crossed lines relative to axis *C* was checked by rotating the sample from 0° to +180° and − 80° about axis *C* and adjusting the position of part two of the sample holder until the laser beam was well centered on the crossed lines in all positions. Part two of the sample holder was then clamped in position.

The position of axis *A* relative to axis *C* was checked by rotating the holder about axis *B* until axis *A* was approximately horizontal, then rotating the sample from 0° to +80° about axis *A.* The position of axis *A* was adjusted by shifting shaft *B* along its axis of rotation until the laser beam was well centered on the crossed lines in all positions. Axis *B* was then fixed in this position.

The above adjustments positioned the various parts of the sample holder relative to the laser beam and to each other so that the laser beam was centered on the point on the sample surface where the three axes of rotation intersected.

### 4.3. Setting Zeros on Each Axis of Rotation

The zero position of each axis was established with a plane mirror in the sample holder. Angle *a* was adjusted as before until the sample was normal to axis *B.* This was the zero position of angle *a.* Angle *c* was then adjusted until the sample was normal to the laser beam. This was the zero position of angle *c*. Angle *c* was then set at about 10°, 40°, 75°, −40°, and −75°, and at each setting a horizontal reference line was drawn on the laboratory wall at a height where the laser spot was bisected by the line. The position of each reference line was checked with the level, to make sure that it was in the reference plane. Angle *c* was then reset at zero, and angles *a* and *b* were adjusted to center the laser spot on one of the reference marks with angle *a* at about ±40°. This was the zero position of angle *b.*

The reference marks on the laboratory wall could be used for a quick check to see that axis *C* was vertical, and to check the zero position of angle *b.*

### 4.4. Aligning Detector Baffles

The detector baffle consisted of several sections of 6 cm i.d. brass tubing of 1.5 mm wall thickness, 5, 10, or 25 cm long. The ends of each tube were squared and smoothed on a lathe. A shoulder 1 mm deep of outside diameter just under 6 cm, was machined on each side of each diaphragm plate, so that it fits snugly into the end of a tube, and could be used as a coupling between tubes. A circular aperture was machined in the center of each plate. The plate was tapered evenly on both sides from about 3 mm from its outer edge to the edge of the aperture, so that the thickness at the edge of the aperture was about 0.25 mm, and the edge of the aperture was rounded. Aperture plates with apertures of various diameters from 1 mm to 6 cm were prepared. The detector was mounted at the center of one face of a brass block of outer diameter such as to fit snugly into the baffle tubing, and the leads were passed through holes in the block. The insides of the tubes, the diaphragms and the end of the block on which the detector was mounted were painted with a diffusely reflecting black paint.

The detector and baffles, in the form of a tube 6 cm in diameter and 75 cm long, rested in four V-blocks mounted on the detector arm of the sample holder. The V-blocks were aligned by use of a 25 cm section of baffle tubing with 2 mm diaphragms in each end. The tube was supported by the first two V-blocks, with angle *c* set at the zero position, and the position of the blocks was adjusted until the laser beam was well centered in the two apertures. The tube was then moved to the second and third V-blocks, and the third block adjusted, then to the third and fourth V-blocks, and the fourth block adjusted. The V-blocks were clamped in position after adjustment.

The detector baffle consisted of lengths of 10, 25, 25, 10, and 5 cm of tubing, with baffle plates at the front end and acting as a coupling between successive tubes. The size of the apertures was graduated for 3 cm at the front end to 1.4 cm in front of the detector so that the view of the detector was restricted to essentially the 3.8 cm hole in the sample holder. The detector was mounted 94 cm from the point where the axes crossed.

### 4.5. Aligning Optical Elements

The mirror chopper consisted of a single sheet of plate glass, cut to have six blades and six spaces, then coated with vacuum deposited aluminum to form a first surface mirror. It is necessary, in order for the reflections from the six blades to coincide, that the mirror be mounted on its rotating shaft so that the plane of the reflecting surfaces is normal to the axis of rotation. Such alignment is difficult to achieve by mechanical means.

The mirror chopper was adjusted until it was nearly normal to the shaft on which it was mounted. The chopper was set in position to reflect the laser beam onto a wall about 3 m away. The position of the beam when reflected by each blade in turn was recorded on a piece of paper. These points fell on a circle. The position of the chopper blade on the shaft was shifted, and the points recorded, until the diameter of the circle had been reduced to about 1 mm. The chopper was then positioned about 18 cm in front of the laser mirror, tilted slightly so that the laser beam was reflected slightly downward to a plane mirror, which reflected it to the entrance aperture of the averaging sphere in front of the reference detector. The plane mirror was adjusted to center the beam in the aperture.

The spatial filter consisted of a microscope objective lens which focused the laser beam onto a 20 *µ*m aperture and a second microscope objective which recollimated the beam emerging from the aperture. The aperture was in a magnetized steel mount which was held magnetically in contact with the aperture plate and with horizontal and vertical micrometer screws. In order to avoid deviation or displacement of the laser beam, it was necessary that the beam be accurately centered on each of the lenses and the aperture. The filter was mounted on a micromanipulator which provided vertical and horizontal adjustment in a plane normal to the laser beam.

The lenses and aperture were removed from the filter, and it was approximately centered and aligned by eye. The aperture was then inserted, and approximately centered in the laser beam. A piece of white cardboard was mounted a few cm behind the aperture, and was uniformly but faintly illuminated by light passing through the aperture. The first lens was then inserted, and brought toward focus, until the card was dark on one side. The aperture was then moved perpendicular to the laser beam toward the light side of the card until the circle of light was again uniform. This process was repeated until the laser beam was fully focused on the aperture, which was indicated by a marked increase in brightness on the card. The focus adjustment was critical, and the focus could be destroyed by movement of only a few micrometers of either the lens or aperture. The second lens was then inserted and the laser beam was recollimated to form a uniform bright spot about 2 mm in diameter on the sample. However, this spot was no longer centered on the crossed lines, but deviated from the cross by several cm. The aperture was removed from the filter, leaving the micrometer screws set at the focus position, and the filter was adjusted in angle and position, until the beam was again centered on the cross. The aperture was then replaced, but was no longer in the focus position, and had to be realigned. The readings on the two micrometer screws were recorded, and the aperture was scanned in a raster pattern over an area about 0.25 mm square about the former focus position, until some light could be seen coming through the filter, after which the aperture was moved toward the light side of the pattern until the focus position was again attained.

The polarization rotator consisted of two 8-mm diameter quartz quarter-wave plates, each mounted in a holder that permitted it to rotate in its own plane. When the plane of retardation of the first filter was at 45° to the plane of polarization of the beam, it converted the plane-polarized beam into a circularly polarized beam. This first plate could be rotated manually. The second plate converted the circularly polarized beam back into a plane-polarized beam whose plane of polarization rotated with the plate. This plate was rotated by a stepping motor in synchronization with that used to rotate the sample about axis *B.* This arrangement maintained the angle between the plane of polarization and the plane of incidence constant as the sample was rotated to change the plane of incidence. The holder for the second plate could easily be removed so that the circularly polarized beam could be used when an unpolarized incident beam was desired.

Alignment and adjustment of the rotator involved mounting the two plates so that the beam was well centered on each plate, and was incident at an angle about 1° off normal, so that interreflections between the plates themselves or other optical elements would be avoided, and adjustment of the angular position of the plane of retardation of each plate. The holder was constructed so that the two plates were mounted at an angle of about 1° to each other. After the holder was properly positioned, the second plate was removed, a rotatable polarizing filter was placed in the beam, and the beam was directed to an averaging sphere in front of a detector. The first plate was positioned so that its plane of retardation was at approximately 45° to the plane of polarization of the laser beam on the basis of the marked position on the plate of the plane of polarization. The residual polarization was measured as the difference between the high and low readings of the detector as the polarizing filter was rotated, divided by the sum of the two readings. Fine adjustments were made in the angular position of the first plate until the residual polarization reached a minimum, which was about 1.5 percent. The second plate was then inserted in its holder, and its angular position adjusted until the plane of polarization was vertical. Angle *b* was then set at zero, and the stepping motors that drove the sample about axis *B* and the polarizing filter, respectively were synchronized.

The final step was to insert an aperture, about 2.5 mm in diameter, into the beam between the laser and the chopper. The aperture was placed so that the beam was well centered, and did not touch the edge of the aperture at any point.

## 5. Errors

The possible sources of error associated with reflectance measurements are listed and discussed below.

### 5.1. Solid Angle of Viewing

For a small solid angle, the following expression can be used

$$\omega =h\cdot w/l^{2}$$

where *ω* is the solid angle subtended by an area with height *h* and width *w* and placed *l* distance away. The error caused by the uncertainties Δ*h*, Δ*w*, and Δ*l* in determining *h*, *w*, and *l* can be expressed as

$$\frac{\Delta \omega }{\omega }={\left[{\left(\frac{\Delta h}{h}\right)}^{2}+{\left(\frac{\Delta w}{w}\right)}^{2}+2{\left(\frac{\Delta l}{l}\right)}^{2}\right]}^{1/2}$$ (5.1)

*h* and *w* can be measured to 0.025 mm and *l* to 1 mm. With *h = w*= 10 mm and *l* = 940 mm, Δ*ω*/*ω* = 0.005. The uncertainty in the size of the solid angle subtended by the detector contributes no error to the measured bidirectional reflectance. However, when the bidirectional reflectance is converted to average bidirectional reflectance distribution function (BRDF) the uncertainty in the size of the solid angle contributes directly to the uncertainty in the average BRDF. With a diffuse sample, the measured average BRDF will have an uncertainty of 0.5 percent of its own value.

### 5.2. Incident Level

The irradiance received by the detector from the laser source is six to seven orders of magnitude higher than that from some of the black coatings.

The incident light is measured by using a 3-inch-diameter averaging sphere in front of the detector and the diffuse light reflected from the sample is viewed by the detector directly. The signal produced by the laser source would be

$$S_{0}=I_{0}\cdot T_{0}\cdot G_{0}$$ (5.2)

and the signal produced by the sample would be

$$S_{s}=I_{0}\cdot \rho \cdot G_{s}$$ (5.3)

where *I*_0_ is the total power in the beam from the source; *T*_0_ is the transmittance of the averaging sphere; *ρ* is the bidirectional reflectance of the sample; and *G*_0_ and *G_s_* are the gains used for measuring the source and sample, respectively.

Combining equation (5.2) and (5.3), the reflectance is expressed as

$$\rho =\frac{S_{s}}{S_{0}}\left(T_{0}\cdot G\right)$$ (5.4)

where

$$G=G_{0}/G_{s}$$

The contribution of *T*_0_ and *G* to the uncertainty of the reflectance can be written as

$$\frac{\Delta \rho }{\rho }={\left[{\left(\frac{\Delta T_{0}}{T_{0}}\right)}^{2}+{\left(\frac{\Delta G}{G}\right)}^{2}\right]}^{1/2}.$$ (5.5)

The transmittance of the averaging sphere (sphere A) was determined by employing another averaging sphere (sphere B) of the same size and construction. A signal was first obtained when both spheres were used with sphere B over the detector and sphere A over sphere B. The signal with just sphere B over the detector was then measured. The ratio of these two signals yielded the transmittance of sphere A. Some uncertainties are involved in the measurements. The interreflection between these spheres, although it existed, was very small. The directly irradiated area on the wall of sphere B was larger in the first measurement than that in the second measurement. The uncertainty of the transmittance *T*_0_ of sphere A is thus dependent on the variation of the reflectance of the directly irradiated portion of the wall of sphere B, which is estimated to be 2 percent of its value.

The true ratio of the gains used was determined by feeding a signal from a square wave generator through an attenuator to the preamplifier of amplifier A (fig. 1). The method of determination was designed so that the ratio thus measured was independent of the accuracy of the attenuator. The uncertainty of the ratio was about 0.22 percent of its value. From equation (5.5), the uncertainty in the measurement of the incident level is about 2.2 percent of its value.

### 5.3. Angular Position

In the plane of incidence and assuming *ϕ*_0_ = 0, equations (3.1) to (3.5) can be rewritten as

$$\begin{array}{l} \theta_{r}=a\\ \phi_{r}=+\;90{}^{\circ}\\ \theta_{i}=\left|\;c+a\;\right|\\ \phi_{i}=-\;90{}^{\circ}. \end{array}$$

From section 2.7, *a* is controlled to 0.1° and *c* to 0.045°. Thus, Δ*θ_r_* = 0.1° and Δ*θ_i_* ≃ 0.15°. For a diffuse sample, the contribution to the uncertainty of the reflectance would be negligible.

### 5.4. Linearity

Nonlinearity within one gain setting of amplifier A was determined to be less than 0.2 percent.

### 5.5. Stability

The measurement is made independent of the fluctuations in the laser source by using the monitor detector. However variations in the gain of the measuring and monitor receiver systems should be considered. The detector is slightly temperature sensitive. The temperature of the laboratory is controlled within one degree. The instability of the detection system is estimated to cause uncertainty in reflectance of 0.2 percent of its value.

### 5.6. Polarization

For samples with random roughness distribution, the use of circularly polarized light to replace unpolarized light should not contribute any error to the reflectance determination.

### 5.7. Random Error

The noise is higher for lower reflectance samples. For a sample with 10^−3^ percent reflectance, the noise is 0.1 percent of its value. For a sample with 2 × 10^−4^ percent reflectance, the noise is 1 percent of its value and for a sample with 2 × 10^−5^ reflectance, the noise is 10 percent of its value. The error due to these noise levels could be reduced by taking more data.

### 5.8. Error Budget

For relative measurements the first two factors (solid angle and incident level) could be ignored. Thus for the absolute and relative measurement with reflectance less than 10^−3^ percent, the error budgets are as follows:

**Table:** 

	Absolute (% of its value)	Relative (% of its value)

Solid angle	0.5	
Incident level	2.2	
Angular position	–	–
Linearity	0.2	0.2
Stability	0.2	0.2
Polarization	–	–
Random noise	0.1–10	0.1–10
	
	3.2–13.1%	0.5–10.4%

### 5.9. Performance Tests

**Figure 5.1:**
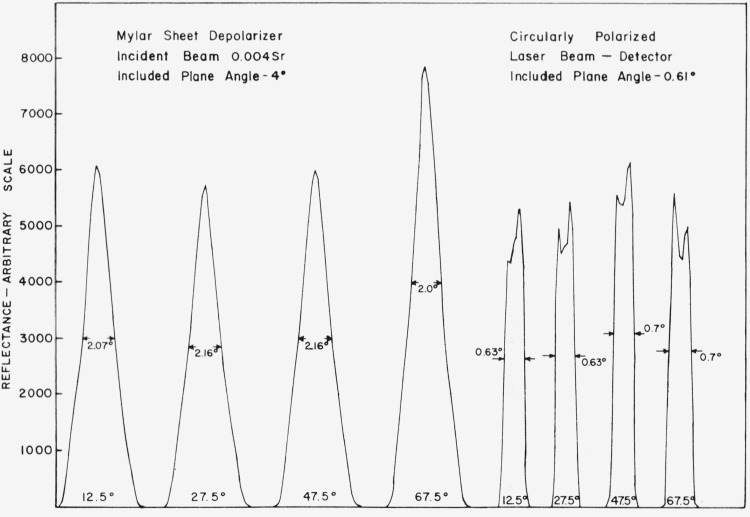
Results of angular resolution tests.

Figure 5.1 shows the angular resolution attained with the instrument. The specular peak reflected by a piece of polished black glass was measured. For the circularly polarized beam the half-height width of the peak is about 0.7 degree, and with the mylar sheet depolarizer the half-height width is about two degrees because the incident beam is convergent.

**Figure 5.2:**
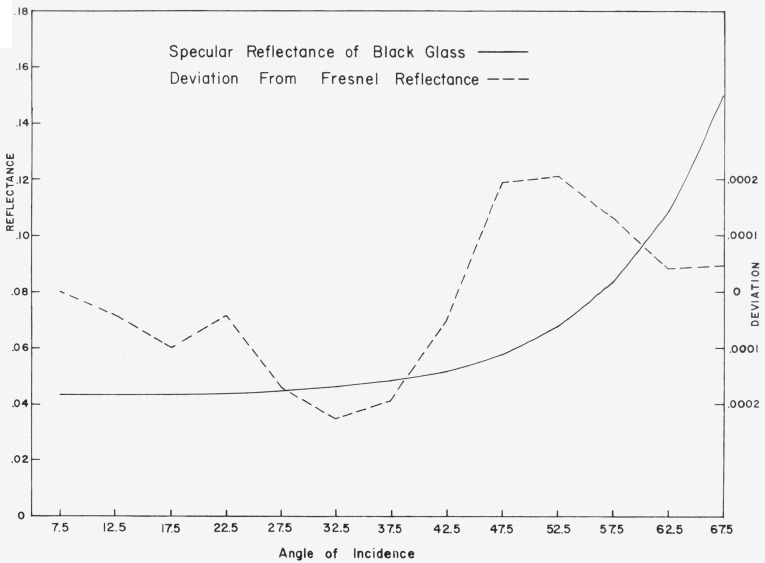
Measured reflectance of black glass compared to reflectance computed from Fresnel’s equation.

The accuracy of the specular measurements is shown in figure 5.2. The absolute reflectance of a piece of polished black glass was measured as a function of angle of incidence. The reflectance was obtained as the ratio of the measured reflected and incident fluxes. The value obtained at 7.5 degrees from the normal was used to compute the index of refraction of the glass, which was in turn used to compute the Fresnel reflectance at the other angles of incidence. The difference between the measured and computed values is shown as the dotted line, and does not exceed 0.02 percent in absolute reflectance.

## 6. Results

**Figure 6.1:**
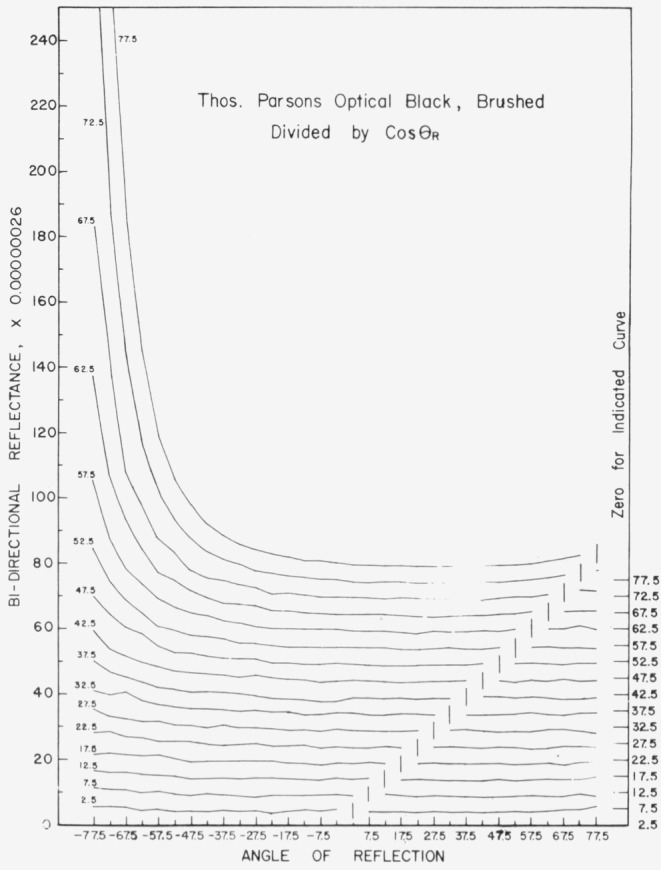
Bidirectional reflectance of brushed Thomas Parsons optical black. For BRDF multiply scale values by 0.0023.

Data for 15 samples of coated metal are shown in figures 6.1 through 6.16. All data were taken at 0.6328 *µ*m in the plane of incidence, at angles of incidence varying from 2.5° to 77.5° from normal at increments of 5°, and at angles of reflection from − 77.5° to + 77.5°, again at increments of 5°. The directions of incidence are defined as positive, hence reflection in a negative direction is forward scattering, and reflection in a positive direction is backscattering.

In order to permit easy comparison of the effects of (1) variations in direction of reflection, (2) variation in direction of incidence, and (3) method of applying a coating, the data were treated in two ways. First, each measured bidirectional reflectance was divided by the cosine of the angle of reflection, *θ_r_*, measured from the normal to the surface to the direction of reflection, in order to show the deviation of the bidirectional reflectance from that of a perfect lambertian diffuser, for which *ρ*/(cos *θ_r_*) would be constant for all angles of reflection. Second, a separate curve was drawn for each direction of incidence. In order to minimize overlap of these curves, the zero line for the incidence at 2.5° from normal was selected to place the curve near the bottom of the graph. The zero line for each succeeding curve was displaced upwards, by an amount indicated on the right hand margin of the graph. Hence for materials that are very good diffusers, the different curves will be essentially flat and parallel, while retroreflectors will exhibit peaks near the direction of incidence, and specular reflectors will exhibit peaks in the specular direction.

The major interest in this study was to develop a method of evaluating bidirectional reflectance, and particularly to study the variations in bidirectional reflectance with directions of incidence and reflection. The coatings chosen for measurement were optical coatings that are either highly absorbant, primarily for use in reducing stray radiation in optical systems, and those that are highly reflecting and good diffusers, for use as integrating sphere coatings.

Since the variations in bidirectional reflectance are of primary interest, the data are plotted in arbitrary units. However, a conversion factor to absolute units is indicated on each figure. The bidirectional reflectance, even of the high-reflectance coatings, is necessarily low, because of the very small solid angle of collection. For many of the coatings, the relative directional hemispherical reflectance was computed from the measured bidirectional reflectance, and plotted as a function of angle of incidence. This graph is shown as an insert in the main figure.

**Figure 6.2:**
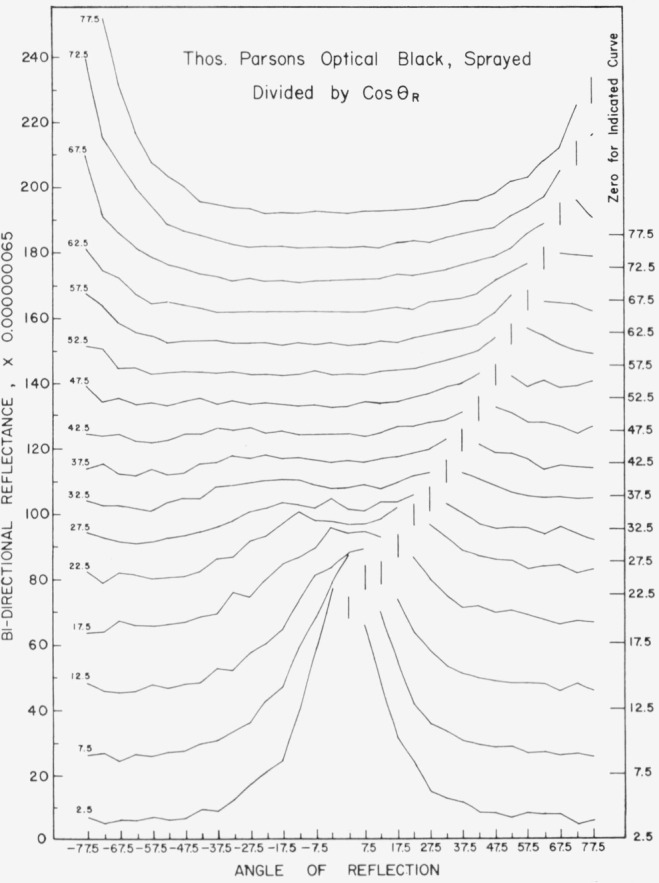
Bidirectional reflectance of sprayed Thomas Parsons optical black. For BRDF multiply scale values by 0.00057.

The bidirectional reflectance of Thomas Parsons Optical Black^2^ applied by brushing is shown in figure 6.1. This coating is very diffuse at angles of incidence within about 30° of normal, and becomes increasingly specular^3^ at increasing angles of incidence beyond 30°. There is no indication of retroreflectance. The same coating applied by spraying, figure 6.2, is a pronounced retroreflector, and somewhat less specular.

**Figure 6.3:**
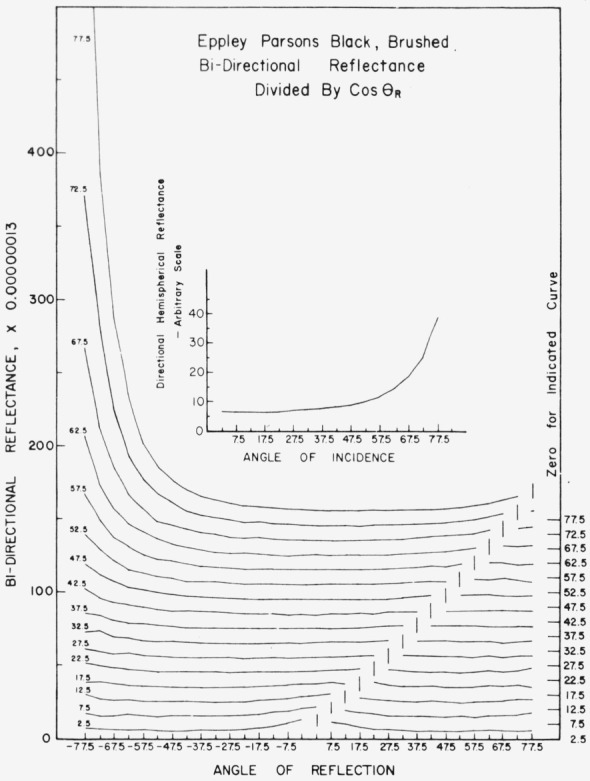
Bidirectional reflectance of brushed Eppley Parsons optical black. For BRDF multiply scale values by 0.00115.

**Figure 6.4:**
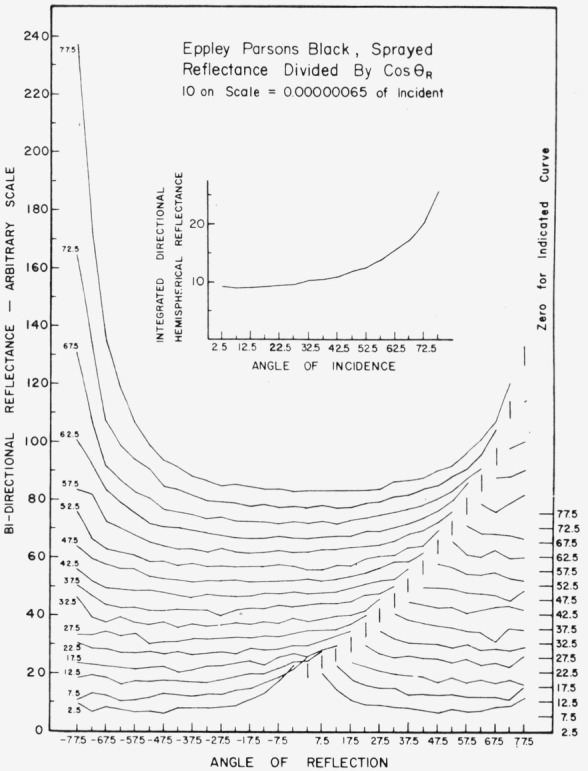
Bidirectional reflectance of sprayed Epply Parsons optical black. For BRDF multiply scale values by 0.00057.

The Eppley Laboratory purchased the rights to this coating from Thomas Parsons in England. Their version of Parsons Optical Black, when applied by brushing, shows in figure 6.3 a slight tendency toward retroreflectance, but in general appears quite similar to the original. When applied by spraying, the Eppley coating shows significantly reduced retroreflection and somewhat greater specular reflection at large angles of incidence, as shown in figure 6.4, compared to the original.

3M Nextel black is very diffuse in reflectance at angles of incidence of 45° or less from normal. At angles of incidence greater than 45° from normal it becomes increasingly specular with an increase in the angle of incidence. There is no pronounced retroreflection.

**Figure 6.6:**
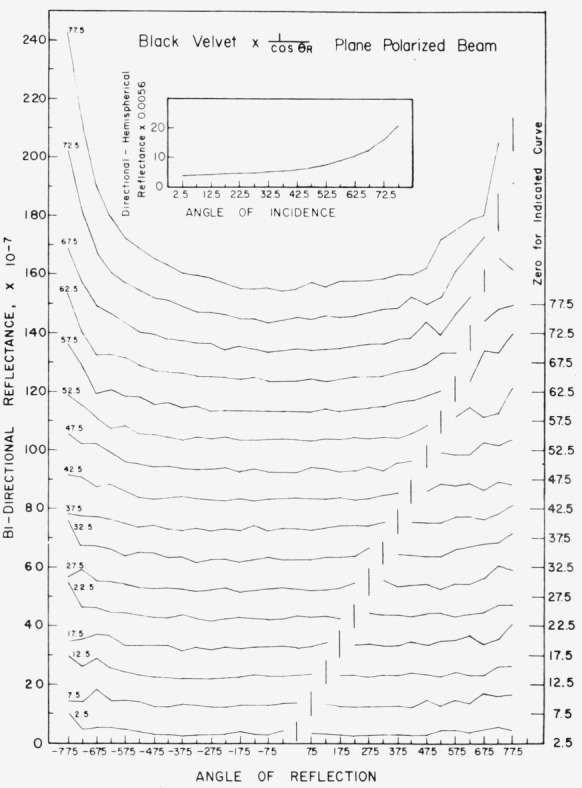
Bidirectional reflectance of black velvet cloth. The incident beam was polarized normal to the plane of incidence. For BRDF multiply scale values by 0.00088.

The data for black velvet cloth, shown in figure 6.6, were taken with a plane polarized incident beam. In all other cases the incident beam was either circularly polarized, or depolarized. The data on black velvet cloth are shown for their general interest. The black velvet is an excellent diffuser, becoming somewhat specular and retroreflective at angles of incidence beyond about 60° from the normal.

**Figure 6.7:**
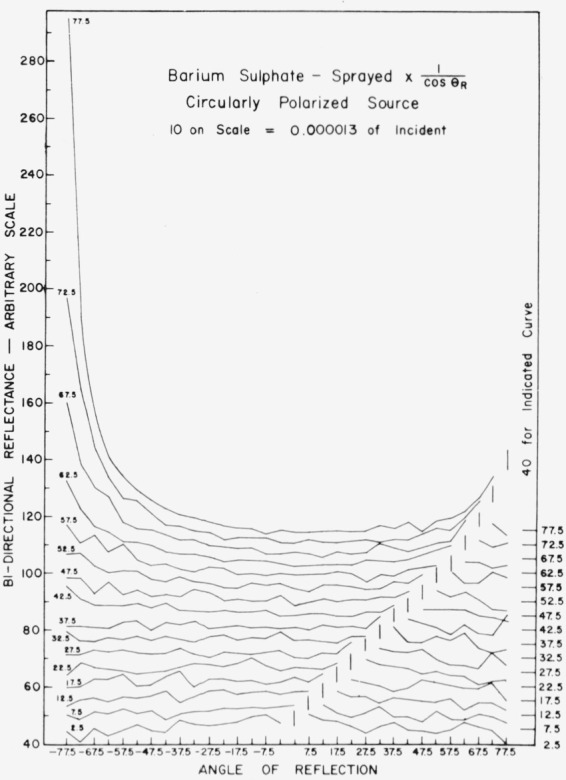
Bidirectional reflectance of sprayed barium sulphate. For BRDF multiply scale value by 0.0115.

**Figure 6.8:**
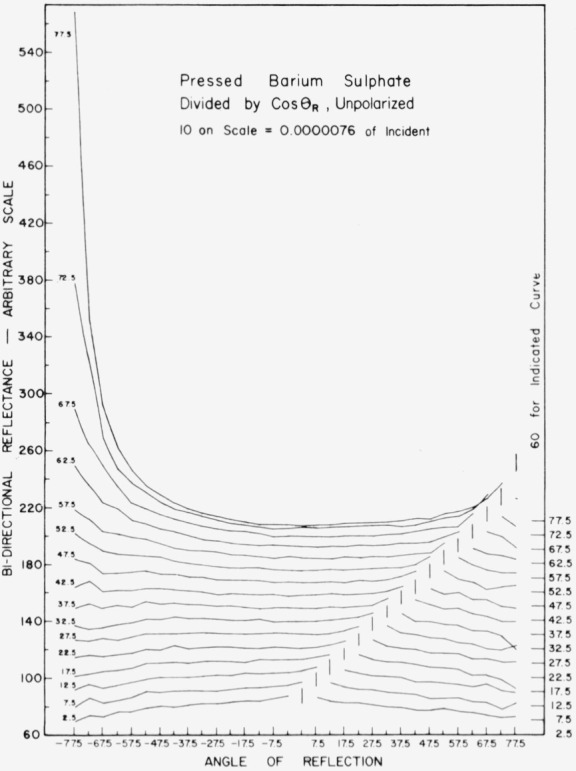
Bidirectional reflectance of pressed barium sulphate. For BRDF multiply scale values by 0.0067.

In the measurements on sprayed barium sulphate, figure 6.7, the incident beam was depolarized by use of the spinning drafting film sheet (see para. 2.5). Sprayed barium sulphate is a good diffuser at angles of incidence less than about 50° from normal. It becomes slightly retroreflective at angles of incidence greater than about 15° from normal, and somewhat specular at angles of incidence greater than about 50° from normal. Even at 77.5° from normal, the retroreflectance is relatively small, but the specular reflection is quite pronounced. The scatter in the data is attributed to roughness of the sprayed coating. The pressed barium sulphate, figure 6.8, is similar to the same coating applied by spraying, but the scatter in the data is greatly reduced, probably because of the smoother surface.

**Figure 6.9:**
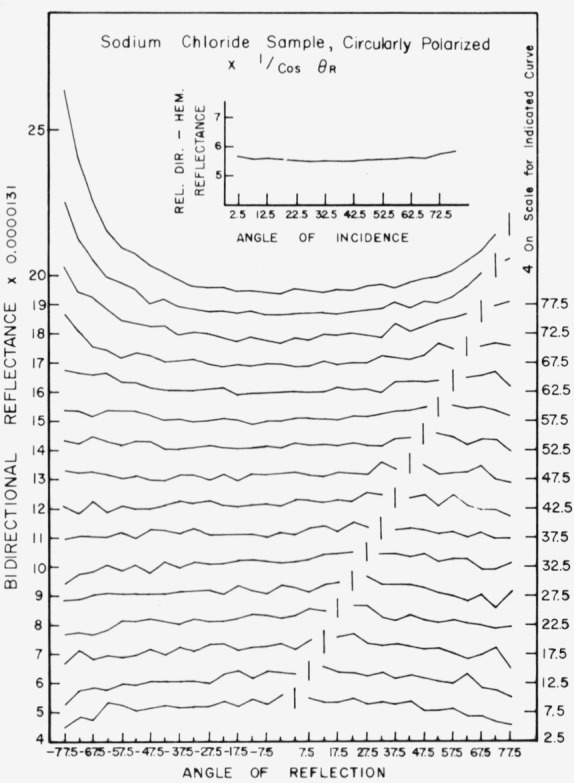
Bidirectional reflectance of sprayed sodium chloride. For BRDG multiply scale values by 0.116.

Sodium chloride, figure 6.9, is an excellent diffuser, showing essentially no specular or retroreflectance at directions of incidence less than about 65° from the normal, and the specular and retroreflectance even at 77.5° from normal is low compared to that of barium sulphate.

**Figure 6.10:**
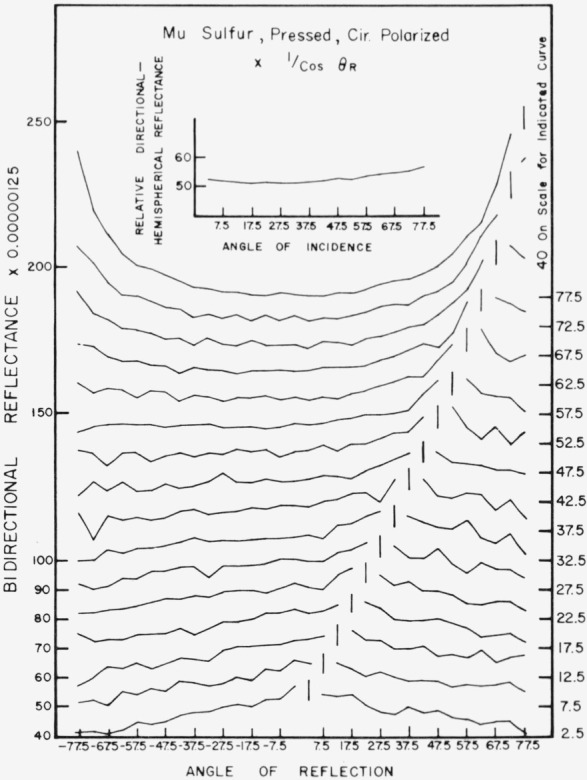
Bidirectional reflectance of pressed Mu sulfur. For BRDF multiply scale values by 0.011.

Mu sulfur, figure 6.10, is also a good diffuser, but becomes somewhat retroreflective at angles of incidence beyond about 15° from normal, and slightly specular at angles of incidence beyond about 65° from normal.

**Figure 6.11:**
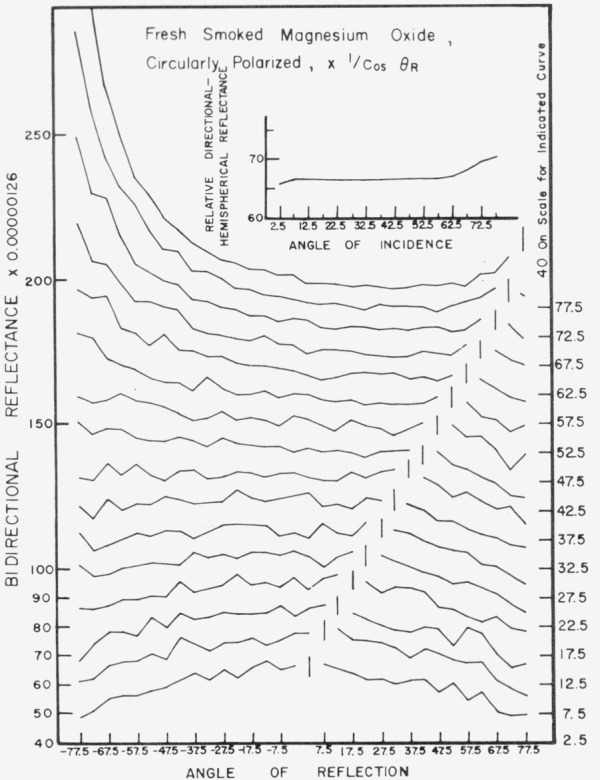
Bidirectional reflectance of smoked magnesium oxide. For BRDG multiply scale values by 0.011.

Smoked magnesium oxide, figure 6.11, is somewhat inferior to barium sulphate and sodium chloride as a diffuser, particularly at angles of incidence near normal. It shows significant fall-off in back reflection with increasing angle of viewing at all directions of incidence.

**Figure 6.12:**
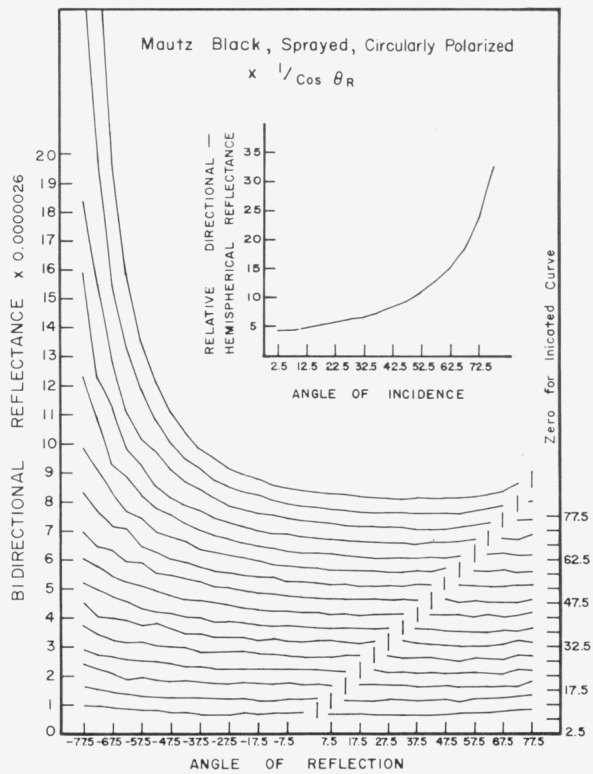
Bidirectional reflectance of sprayed Mautz black. For BRDF multiply scale values by 0.023.

**Figure 6.13:**
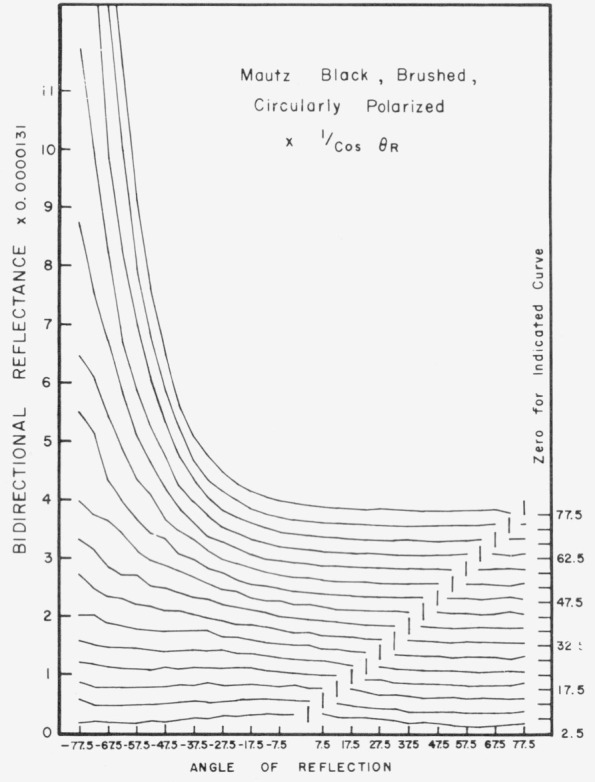
Bidirectional reflectance of brushed Mautz black. For BRDF multiply scale values by 0.116.

Sprayed Mautz black, figure 6.12, is a good diffuser at near normal incidence, but becomes increasingly specular with increasing angle of incidence beyond about 10° from normal, and a slight retroreflector at angles of incidence near grazing. The same coating when brushed, figure 6.13, becomes even more specular, especially at near grazing angles.

**Figure 6.14:**
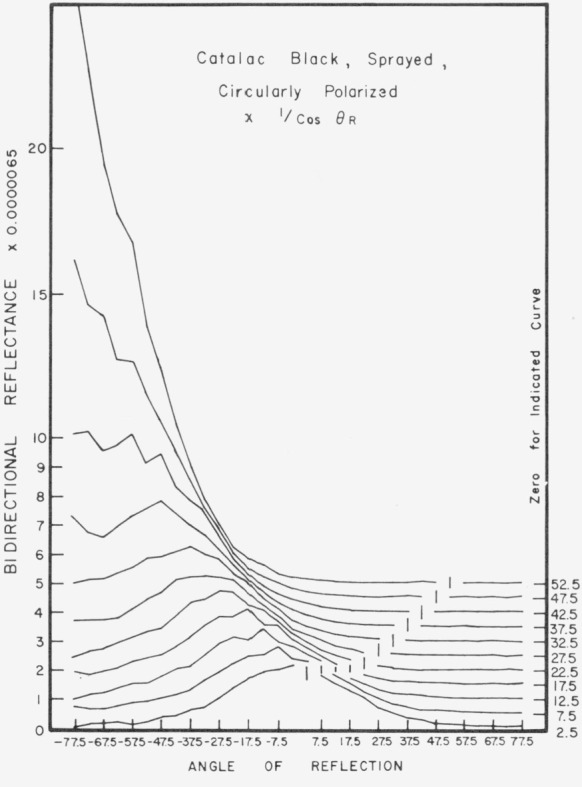
Bidirectional reflectance of sprayed Catalac black. For BRDF multiply scale values by 0.057.

**Figure 6.15:**
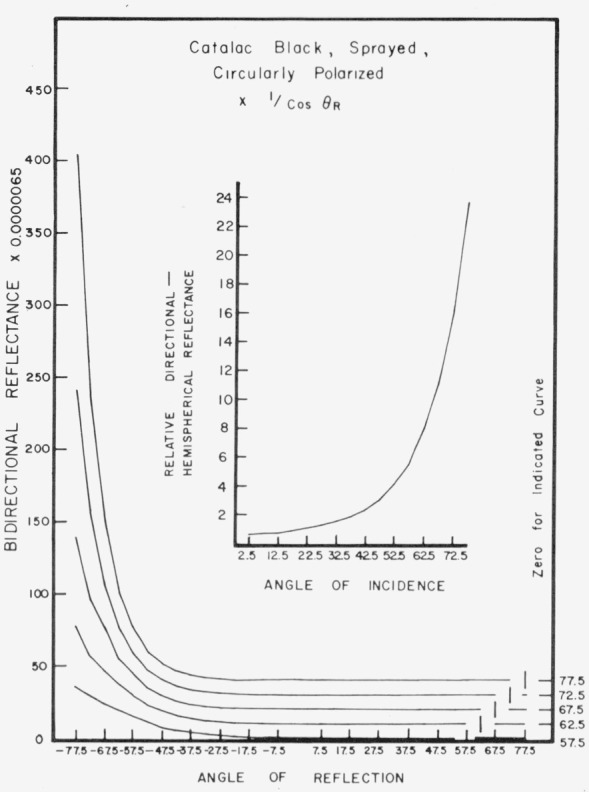
Bidirectional reflectance of sprayed Catalac black. For BRDF multiply scale values by 0.057.

**Figure 6.16:**
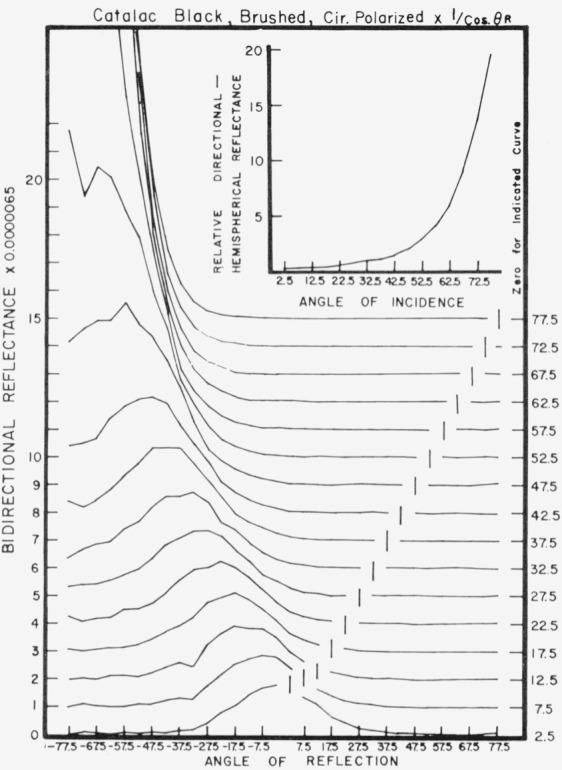
Bidirectional reflectance of brushed Catalac black. For BRDF multiply scale values by 0.057.

Sprayed Catalac black, figures 6.14 and 6.15, shows significant specular reflection even at near-normal directions of incidence, and is very specular at near grazing angles. This coating shows about the same pattern when brushed, figure 6.16.

It should be noted that the specular peak of the diffusely reflecting coatings tends to occur at an angle of reflection greater than the angle of mirror reflection, and for some coatings at an angle of reflection beyond 77.5° from normal. This tendency has been noted by previous workers in the field. [4]

## 7. Conclusion

This paper has described the design, construction and alignment of a laser source bidirectional reflectometer that is fully automated and has angular resolution on the order of one degree. The directions of incidence and viewing can be independently varied over an entire hemisphere except for directions more than 77.5° from the normal, and the two directions must be at least 2.5° apart. Error analyses has been performed. Bidirectional reflectance for 15 samples of black and white coatings are presented.

It is clear in retrospect that many of the design features and procedures could be improved: (1) kinematic design to allow simpler alignment procedure; (2) designs that can measure liquid and loose powder sample; (3) designs that can measure bidirectional transmittance over an entire hemisphere; (4) better attenuation method to obtain higher accuracy in absolute reflectance determination; and (5) tunable laser or continuous wavelength source to allow wider wavelength range.

An extensive bibliography on scattering by reflection from surfaces, in which the papers are classified by subject matter, immediately follows this paper.

## Figures and Tables

**Figure 2.3A:**
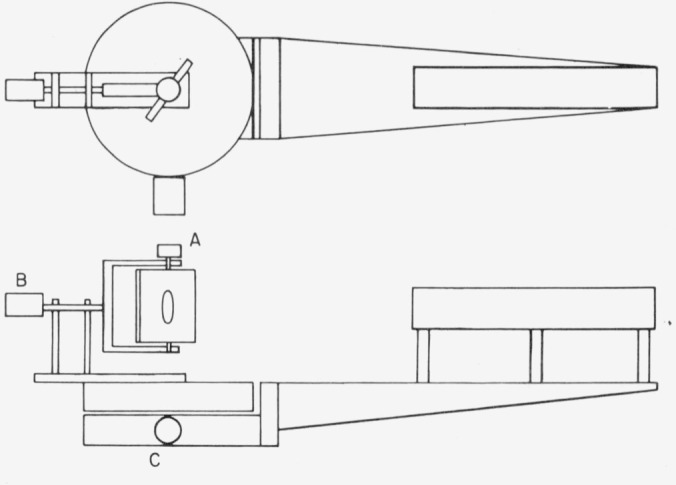
Schematic drawing of sample holder and turntables. A, B, and C are stepping motors.

**Figure 2.3B:**
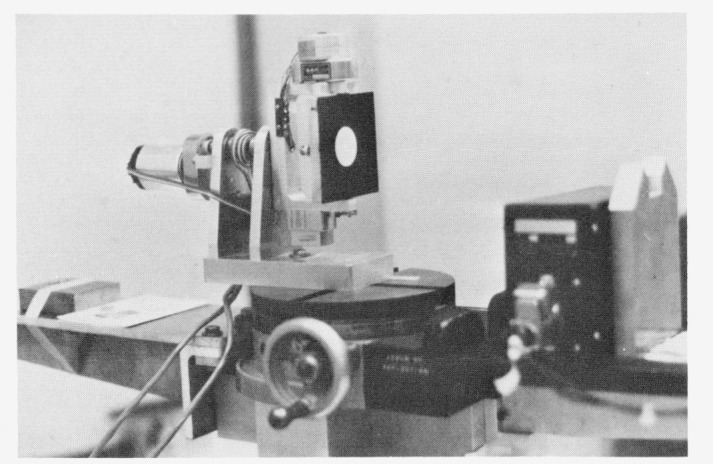
Photograph of sample holder.

**Figure 6.5:**
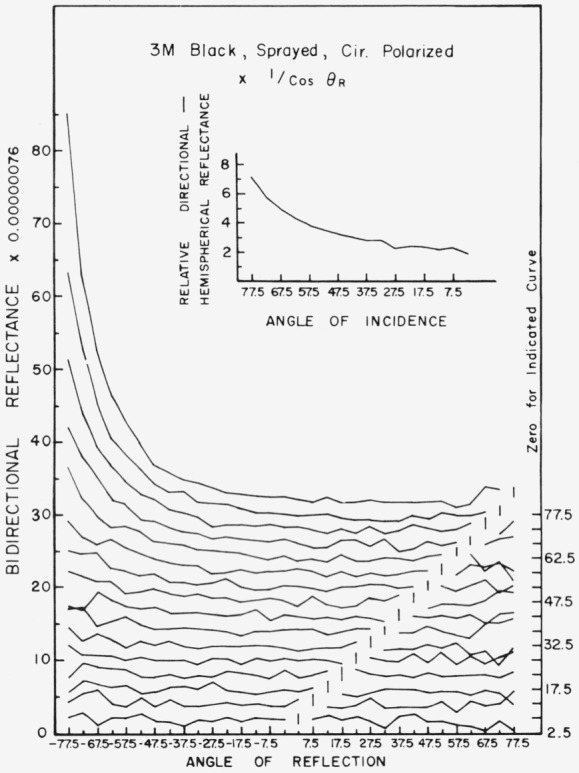
Bidirectional reflectance of sprayed 3M black. For BRDF multipy scale values by 0.0067.

## Footnotes

1 Figures in brackets indicate the literature references at the end of this paper.

2 The proprietary coatings measured in this study are identified by manufacturer and trade name for the convenience of the reader. Such identification does not constitute endorsement of the coatings by the National Bureau of Standards.

3 But not at the normal angle for specular reflection. See discussion at end of this section.
